# Supplementary material for: Detection of EGFR Mutations in Plasma Cell-Free Tumor DNA of TKI-Treated Advanced-NSCLC Patients by Three Methodologies: Scorpion-ARMS, PNAClamp, and Digital PCR
Source: Diagnostics (Basel). 2020 Dec 7;10(12):1062. doi: 10.3390/diagnostics10121062 (PMC7762356; doi:10.3390/diagnostics10121062)
Supplement: Supplementary file 1 [file diagnostics-10-01062-s001.zip › diagnostics-1001893_Table S1.pdf]

Table 1. Number of EGFR mutated copies as quantified by QuantStudio 3D Digital PCR assay at baseline. The amount of the three main *EGFR* mutations in cell-free DNA is reported as copied/ul of plasma (ND, not determined; WT, wild type for the specific mutation). Results in the table are referred to Figure 2.

Patient ID	T790M	Exon 19 deletion	L858R
1	0.423	WT	WT
2	0.381	0.774	WT
3	ND	ND	ND
4	WT	0.416	WT
5	0.938	WT	WT
6	WT	WT	0.417
7	WT	0.379	WT
8	ND	ND	ND
9	0.374	0.447	WT
10	WT	5.938	WT
11	WT	201.07	WT
12	WT	171.75	WT
13	WT	4.047	WT
14	WT	2.888	WT
15	WT	0.481	WT
16	WT	12.221	WT
17	ND	ND	ND
18	WT	0.36	WT
19	ND	ND	ND
20	ND	ND	ND
21	WT	0.408	WT
22	ND	ND	ND
23	1.69	WT	WT
24	2.826	WT	3916.4
25	WT	0.442	WT

26	WT	17.53	WT
27	ND	ND	ND
28	16.092	WT	14.023
29	WT	0.466	WT
30	WT	153.15	WT
31	ND	ND	ND
