# Supplementary material for: Detection of EGFR Mutations in Plasma Cell-Free Tumor DNA of TKI-Treated Advanced-NSCLC Patients by Three Methodologies: Scorpion-ARMS, PNAClamp, and Digital PCR
Source: Diagnostics (Basel). 2020 Dec 7;10(12):1062. doi: 10.3390/diagnostics10121062 (PMC7762356; doi:10.3390/diagnostics10121062)
Supplement: Supplementary file 1 [file diagnostics-10-01062-s001.zip › diagnostics-1001893_Table-S2.pdf]

	T790M-DIGITAL PCR (COPIES/µl)					DEL19-DIGITAL PCR (COPIES/µl)					L858R-DIGITAL PCR (COPIES/µL)				
**Patient ID**	**T0**	**T8**	**T20**	**EV**	**PD**	**T0**	**T8**	**T20**	**EV**	**PD**	**T0**	**T8**	**T20**	**EV**	**PD**
**1**	0.423	0	0	0	ND	0	2.166	0	0.572	ND	ND	ND	ND	ND	ND
**2**	0.381	0	0.382	0.387	0.793	0.774	0	0.373	0.42	2,077	ND	ND	ND	ND	ND
**3**	0	0	0	0.385	ND	ND	ND	ND	ND	ND	ND	ND	ND	ND	ND
**4**	0	0.39	0	0.388	ND	0.416	0	0	0	ND	ND	ND	ND	ND	ND
**5**	0.938	0	0	1.091	1.957	ND	ND	ND	ND	ND	0	1.157	0.361	0	0
**6**	0	0	0	0	0.382	ND	ND	ND	ND	ND	0.417	0.41	0	0	0.376
**7**	0	0	0	0.383	0	0.379	0	0	0	0	ND	ND	ND	ND	ND
**8**	0	0	0.397	ND	0	ND	ND	ND	ND	ND	0	0.392	0		1.871
**9**	0.374	0	0	0	1.664	0.447	1.105	0	0	1.579	ND	ND	ND	ND	ND
**10**	0	0	0	0	0.812	5.938	0.365	1.201	0	3,055	ND	ND	ND	ND	ND
**11**	0	0	0	0	1.656	201.07	2.248	0	0	10.307	ND	ND	ND	ND	ND
**12**	0	0	0	0	ND	171.75	3.612	0	0	ND	ND	ND	ND	ND	ND
**13**	0	0	0	0	ND	4.047	0.384	0	0	ND	ND	ND	ND	ND	ND
**14**	0	0	0	0	0	2.888	0	0.532	0.386	1.602	ND	ND	ND	ND	ND
**15**	0	0	0	0	0.432	0.481	0	0	0	0.422	ND	ND	ND	ND	ND
**16**	0	0	0	0	2.009	12.221	5.707	0.393	0	33.686	ND	ND	ND	ND	ND
**17**	0	0	0	0	0	0	0.934	0	0	0	ND	ND	ND	ND	ND
**18**	0	0	0	0	0.382	0.36	0.522	0	0	0.363	ND	ND	ND	ND	ND
**19**	0	0	0	0	ND	0	0	0	0	ND	ND	ND	ND	ND	ND
**20**	0	0	0	0	ND	ND	ND	ND	ND	ND	ND	ND	ND	ND	ND
**21**	0	0	0	0	ND	0.408	0	0	0	ND	ND	ND	ND	ND	ND
**22**	0	0	0	0	ND	ND	ND	ND	ND	ND	0	0	0	0	ND
**23**	1.69	0	0	0	ND	ND	ND	ND	ND	ND	ND	ND	ND	ND	ND
**24**	2.826	0.455	0	0.473	99.902	ND	ND	ND	ND	ND	3916.4	20.862	4.668	0.732	319.94
**25**	0	ND	0	0	0	0.442	ND	0	3.56	0	ND	ND	ND	ND	ND
**26**	0	0.436	0.409	0.81	0	17.53	1.615	0	0	0.468	ND	ND	ND	ND	ND
**27**	0	0	0	0	ND	ND	ND	ND	ND	ND	0	0	0	0	ND
**28**	16.092	3.229	8.53	49.664	0.768	ND	ND	ND	ND	ND	14.023	2.627	13.717	38.613	0.859
**29**	0	0	0	0	ND	0.466	1.183	0.354	0	ND	ND	ND	ND	ND	ND
**30**	0	0	0	0	ND	153.15	3.083	0	0	ND	ND	ND	ND	ND	ND
**31**	0	0	0	0	0	ND	ND	ND	ND	ND	ND	ND	ND	ND	ND

T0: baseline

T8: 8 days after treatment

T20: 20 days ater treatment

EV: first clinical evaluation

PD: progressive disease

ND: not determined
